# Supplementary material for: Are carbon emissions trading and green financial instruments synergistic? -Comprehensive quantitative research based on content analysis
Source: PLoS One. 2024 Mar 7;19(3):e0298601. doi: 10.1371/journal.pone.0298601 (PMC10919601; doi:10.1371/journal.pone.0298601)
Supplement: S1 Data — (ZIP) [file pone.0298601.s001.zip › Data/地级市Z_F工作报告.zip!/┴╔─■/╠·┴δ╩╨/└┤╫╘╣1⁄2╓┌║┼í╛┬φ┐╦╩2╛▌═°í┐_2018.docx]

各位代表：

现在，我代表市人民政府向大会报告工作，请予审议，并请市政协委员和列席同志提出意见。

一、过去五年及2017年工作回顾

市七届人大一次会议以来的五年，是铁岭发展史上极不寻常的五年。我们在省委、省政府和市委的坚强领导下，认真学习贯彻党的十九大和习近平总书记系列重要讲话精神，深入落实新发展理念和“四个着力”“三个推进”，积极应对经济持续下行、矛盾风险集中显现和政治生态净化修复等各种复杂因素的影响，坚定不移推进全面从严治党，统筹抓好改革发展稳定各项工作，保持了全市经济社会平稳发展。

国民经济筑底企稳。坚持实事求是原则，对各项主要指标数据全面做实。在连续23个月大幅下降的情况下，地区生产总值、一般公共预算收入等指标实现了正增长。固定资产投资降幅逐年收窄，城乡居民收入持续增加，经济发展逐步回归正常轨道。

产业结构不断优化。新型农业经营主体快速增长，农业综合生产能力明显提升。新能源产业发展壮大，地方工业体系逐步建立，工业经济总体运行平稳。省级服务业集聚区建设稳步推进，电子商务、金融等现代服务业快速发展，第三产业增加值占地区生产总值的比重达到47%。

城乡环境得到改善。实施了棚户区、弃管楼、农村危房改造和保障性住房建设，市政设施改造有序推进，哈大客专、沈四高速、新三线、光荣街南段等重点交通工程竣工通车。生态治理工程深入实施，一批突出问题得到有效解决，人民群众生产生活环境进一步改善。

各项改革逐步推进。“放管服”改革不断深化，公共行政服务四级联动平台实现全覆盖。高新区、凡河新区管理体制得到理顺。政府机构改革加快推进，机关公务用车改革基本完成，农村综合改革进展顺利，国资国企、财税体制、政府债务管理等其他改革稳步实施。

民生保障日臻完善。每年为百姓办好一批民生实事。脱贫攻坚取得阶段性成果。就业创业工作积极推进，社会保障水平逐年提高。义务教育均衡发展取得新进展。城乡公共文化服务体系逐步健全。基层医疗卫生服务体系基本建成。平安铁岭建设扎实开展，社会治理全面加强，社会保持和谐稳定。

各位代表：刚刚过去的2017年，全市上下积极应对挑战，奋力攻坚克难，经济社会呈现稳中向好的发展态势。预计，地区生产总值完成610亿元，增长4%；一般公共预算收入49.1亿元，增长5.9%；固定资产投资105亿元，下降14.1%；规模以上工业增加值115亿元，增长7%；社会消费品零售总额437.4亿元，增长2%；外贸出口总额11.3亿元，增长27%；城乡常住居民人均可支配收入实现23200元和13410元，分别增长6.5%和7%。

—— 积极推进供给侧结构性改革，产业结构进一步优化。省政府下达的“三去一降一补”任务全部完成，关闭地方煤矿8处，完成煤炭去产能87万吨。粮食总产量实现92.4亿斤，肉类总产量53万吨，榛子等特色产业快速发展，规模以上农业产业化企业总产值实现300亿元。铁岭县农业科技示范园区通过国家验收。省级开发区实现县域全覆盖，3个产业集群销售收入突破10亿元，

新增销售收入超亿元企业7户，实施嫁接改造项目21个、产学研合作项目60项，高新技术企业发展到58户，全市有效发明专利达到374件。省级服务业集聚区在建亿元以上项目10个，建成现代物流配送中心6个。开原网贸港、铁岭县农村淘宝等电商平台和企业经营形势向好，调兵山联睿科技服务外包项目投入运营。3家企业在“新三板”及省级股权交易中心挂牌。新增域外金融机构2家，昌图县农商行完成筹建，金融机构存贷款余额分别增长6.4%和7.4%。全市旅游业总收入增长11.4%。

—— 大力开展招商引资，项目年活动取得新进展。全市到资项目179个，到位资金83.3亿元。开复工亿元以上项目102个。向上争取各类政策资金137亿元。马迭尔冬宫等一批项目达成合作协议，大连汇能、铁煤建工越南工程等一批项目签约。30万吨燃料乙醇、牧原生猪产业化等一批项目开工，西丰茅台白酒、中利渔光互补等一批项目投产。全域化老工业区搬迁改造全面启动，13个独立工矿区搬迁改造项目全部开工。铁岭保税物流中心获国家海关总署批复，铁路物流基地筹建运营。“铁四通辽”经济协作区规划开始编制，与淮安对口合作协议正式签署。

—— 加大基础设施建设力度，城乡环境进一步优化。“多规合一”工作全面启动。实施中心城区重点建设工程15项，城市精细化管理水平明显提升。县城综合承载能力不断增强，西丰县获批省级新型城镇化试点县。杨木林子等4个乡镇进入省特色乡镇培育行列。建设美丽乡村示范村30个。改造棚户区住房1253套、弃管楼182栋、农村危房2721户。建设一事一议村内道路607.7公里，新建和维修各级公路746.2公里。沈康高速调兵山连接线路基工程完工。新建和改造66千伏变电站9座，线路长79.8公里。

—— 加强环境保护工作，生态文明建设取得新进展。中央环保督察反馈意见有效落实。拆除10吨以下燃煤小锅炉605台，20吨以上燃煤锅炉实现稳定达标排放。淘汰黄标车和老旧车辆5237台，上线新能源公交车210辆。中电环保垃圾发电项目开工建设。市县乡三级河长制全部建立。中心城区河湖连通生态治理一期工程竣工，亮子河、马仲河污染防治工作全面开展。全年植树造林20.7万亩，辽河干流铁岭段生态封育18.2万亩。创建省级生态乡镇1个、生态村13个。

—— 全面深化各项改革，发展活力逐步增强。取消调整行政职权事项248项，建立了审批代办帮办和容缺受理机制。工商登记实现多证合一，新增市场主体2.3万户。国有企业集团组建工作启动实施，20户国有企业“三供一业”分离移交工作顺利完成。农村土地流转面积居全省首位，完成确权登记434万亩，发放“两权”抵押贷款4.7亿元。调兵山市被确定为全国推进农村集体产权制度改革试点县。城市公立医院改革全面启动，药品加成全部取消。公证体制、供销社等其他各项改革稳步推进。

—— 切实增进民生福祉，各项社会事业繁荣发展。年初确定的15件民生实事全部落实。28537名建档立卡贫困人口稳定脱贫，63个贫困村销号。新增城镇就业1.4万人。社保提标政策全面落实，养老金按时足额发放。新农合和大病保险实现“一站式”报销，跨省异地就医实现医保支付直接结算。新建连锁幼儿园60所，57所义务教育薄弱学校完成改造，铁岭县、西丰县通过全国义务教育发展基本均衡县认定。铁岭师专现代服务业专业群获批辽宁省高水平特色建设项目。铁岭技师学院被授予全国职业教育培训示范点，培训的选手获得世界技能大赛金奖。综合性老年人养护中心开工建设。完成贫困残疾人家庭无障碍改造1150户。家庭医生签约服务重点人群覆盖率达到60%。各项文化体育活动成功举办，2部小品小戏作品获国家艺术基金支持，2部微电影获亚洲微电影节金海棠奖。我市运动员宋布寒获男子气步枪世界杯冠军。获评全国文明单位和文明村6个。

—— 深入推进依法行政，社会保持和谐稳定。坚持每季度向市委报告政府党组工作。主动接受市人大及其常委会的法律监督、工作监督和市政协民主监督。认真听取各民主党派、工商联和无党派人士意见。高质量办理人大议案、人大代表建议和政协提案438件。“两学一做”学习教育常态化制度化深入开展，政府系统全面从严治党各项工作扎实推进。中央和省委巡视反馈意见有效整改。督查考评、应急管理、政务公开工作不断加强。中茂步行街等一批历史遗留问题逐步化解，市长服务热线和民心网诉求办理工作居全省前列。城乡公共法律服务体系初步建成，法律援助咨询服务实现全覆盖。调兵山市被确定为国务院行政执法三项制度试点地区，市司法局被评为全国人民调解宣传工作先进集体。平安铁岭建设取得新成效，安全生产网格化管理经验在全省推广。

民族、宗教、审计、外事、侨务、人防、农机、气象、地震、国家安全、档案、烟草、检验检疫、武警、消防、国防动员、驻外联络、市志、仲裁等工作也都取得了新成绩。

各位代表：在总结工作的同时，我们还要清醒地看到，全市经济社会发展面临的困难和挑战。一是经济总量仍然较小，发展质量和水平不高，区域、城乡发展不平衡不充分问题还比较突出。农业资源优势没有得到充分发挥，能源工业“一业独大”的局面没有根本转变，现代服务业发展水平不高，战略性新兴产业等新动能还没有形成支撑力量。二是政府职能转变还不到位，制约发展的体制机制问题亟待破解。服务企业和群众“最后一公里”问题还没有很好解决，营商环境仍需进一步改善。三是各类矛盾集中显现，风险防控压力巨大。财政收支矛盾突出，养老金缺口大，地方政府性债务负担沉重。地方法人银行业金融机构风险依然存在。一批信访积案化解和生态环境突出问题治理难度较大。保障和改善民生任务依然繁重。社会治理能力还需进一步提高。四是政治生态净化和修复工作任重道远。少数干部存在能力不足、懒政怠政、推诿扯皮现象，一些领域腐败问题时有发生，形式主义、官僚主义、不担当不作为、做表面文章等问题还程度不同地存在。我们一定要直面问题、迎接挑战，勇于创新、敢于担当，下大力气用改革创新的举措和发展的办法，着力解决这些矛盾和问题，发扬钉钉子精神，久久为功，决不辜负党和人民的重托。

各位代表：走过五年的风雨历程，我们深刻认识到，只有坚持党的领导，旗帜鲜明讲政治，牢固树立“四个意识”，坚决维护以习近平同志为核心的党中央权威和集中统一领导，才能始终走在正确的发展道路上。我们深刻认识到，只有坚决贯彻新发展理念，认真贯彻“四个着力”“三个推进”，牢牢把握供给侧结构性改革这条主线，坚持稳中求进工作总基调，抓住主要矛盾，明确主攻方向，才能不断取得振兴发展的新突破。我们深刻认识到，只有树立抓项目就是抓发展、抓转型、抓后劲的理念，坚定不移实施项目驱动战略，持续开展项目年活动，抓好有效投资，大力推进改革创新，才能获得振兴发展的持久动力。我们深刻认识到，只有坚持以人民为中心的发展思想，着力提高保障和改善民生水平，全面推进社会事业发展，加强社会治理体系和治理能力建设，才能带领人民不断创造美好生活。我们深刻认识到，只有牢固树立正确的政绩观，始终做到“三严三实”“讲诚信、懂规矩、守纪律”，多干打基础、利长远的事，真抓落实、精准落实、有效落实，才能取得实实在在的发展成效。我们深刻认识到，只有认真落实全面从严治党主体责任，严格遵守党章党规党纪，筑牢拒腐防变思想防线，始终做到依法用权、秉公用权、阳光用权、廉洁用权，才能换来铁岭的海晏河清、朗朗乾坤。

各位代表：五年的发展历程波澜起伏、不同寻常，当前的发展形势来之不易、弥足珍贵。这是省委、省政府和市委坚强领导的结果，是市人大及其常委会法律监督、工作监督和市政协民主监督与大力支持的结果，更是全市广大干部群众齐心协力、共同奋斗的结果。在此，我代表市人民政府，向辛勤工作在各条战线上的全市各族人民，向积极支持政府工作的市人大代表、市政协委员、各民主党派、工商联、无党派人士、各人民团体、中省直驻铁单位和各界人士，向驻铁部队和武警官兵，向所有关心、支持、参与铁岭振兴发展的海内外朋友致以崇高的敬意！

二、今后五年政府工作的总体要求和目标任务

今后五年，是实现“两个一百年”奋斗目标的历史交汇期，也是铁岭全面建成小康社会、实现振兴发展的攻坚期。党的十九大描绘了决胜全面建成小康社会、夺取新时代中国特色社会主义伟大胜利的宏伟蓝图，为我们指明了发展方向、提供了根本遵循。省委、省政府实施突破辽西北、县域经济发展等“五大区域发展战略”，为我们进一步理清了发展思路、明确了奋斗目标。市委、市政府制定了《加快突破辽西北 决胜全面建成小康社会三年攻坚计划》，为未来发展确定了重点任务、制定了保障措施。特别是全市风清气正的从政环境和山清水秀的政治生态正在形成，300万人民谋发展、促振兴的愿望愈加强烈。只要我们紧密团结在以习近平同志为核心的党中央周围，坚持和依靠党的领导，增强“四个意识”，坚定“四个自信”，主动适应和引领经济发展新常态，抓住中央和省重大战略机遇，以永不懈怠的精神状态和一往无前的奋斗姿态，汇聚起全市人民的积极性和创造力，紧盯目标、埋头苦干，就一定能够创造出无愧于党、无愧于人民、无愧于时代的工作业绩。

今后五年政府工作的总体要求是：全面贯彻落实党的十九大精神，以习近平新时代中国特色社会主义思想为指导，坚持稳中求进工作总基调，坚持新发展理念，紧扣我国社会主要矛盾新变化，按照高质量发展要求，统筹推进“五位一体”总体布局，协调推进“四个全面”战略布局，深入落实“四个着力”“三个推进”，以供给侧结构性改革为主线，按照省委、省政府的决策部署，认真实施“一带五基地”建设和“五大区域发展战略”，突出开放牵动、创新引领、改革推动、民生优先、绿色发展，坚决打赢防范化解重大风险、精准脱贫、污染防治三大攻坚战，努力走出一条更高质量、更有效率、更加公平、更可持续的发展新路，如期全面建成小康社会，为开创铁岭振兴发展新局面奠定坚实基础。

今后一个时期，我们的奋斗目标是：到2020年，与全省同步全面建成小康社会；在此基础上，再奋斗十年，到2030年实现铁岭全面振兴。未来五年的主要目标是：

—— 经济发展水平明显提高。地区生产总值、一般公共预算收入、固定资产投资等主要经济指标增速不低于全省平均水平，在实际工作中争取更好结果。经济总量进一步扩大，经济发展质量和效益逐年提升。

—— 创新发展成效明显提升。重要领域和关键环节改革实现新突破，供给侧结构性改革取得新进展，经济发展内生动力明显增强。科技贡献率大幅提升，企业创新主体地位更加突出，大众创业、万众创新活力持续迸发。

—— 协调发展能力持续增强。区域发展布局日趋优化，乡村振兴战略深入实施，县域经济实力显著增强。对外开放水平不断提升，经济建设与社会建设统筹推进，发展不平衡不充分问题逐步得到有效解决。

—— 生态建设实现新发展。生态文明理念深入人心，蓝天、碧水、青山、沃土、农村环境综合治理工程扎实推进，生态文明制度体系基本建立，生态环境监管体制基本形成，美丽铁岭建设取得明显成效。

—— 社会文明达到新高度。精神文明建设扎实推进，文化事业和文化产业更快发展。城乡居民思想道德素质、科学文化素质和健康素质普遍提升，全社会文明意识、诚信意识、法治意识不断增强。

—— 人民生活水平普遍提高。基本公共服务均等化水平逐步提升，城乡居民人均可支配收入增速不低于经济增长水平。建档立卡贫困人口全部脱贫，贫困村全部销号，西丰贫困县如期摘帽。社会治理能力明显增强，全市社会保持和谐稳定。

为实现上述目标，今后五年的战略任务是：

1. 坚持创新发展，在培育发展新动能上实现新突破。把创新作为引领发展的第一动力。深入推进供给侧结构性改革，发展壮大传统优势产业，大力发展战略性新兴产业，着力提高供给体系质量。建立科技创新体系，完善创新载体，提升创新能力，引导创新要素向实体经济集聚。实施增品种、提品质、创品牌专项计划，推动铁岭产品向铁岭品牌转变。大力实施人才兴市战略，推动大众创业、万众创新。全面推进重点领域和关键环节改革，破除体制机制障碍，为全市振兴发展创造最优环境。

2. 坚持协调发展，在解决发展不平衡问题上实现新突破。正确处理发展中的重大关系，推进“四化”融合，统筹城乡、区域发展，增强经济和社会发展的整体性、协调性。主动对接“一带五基地”建设和“五大区域发展战略”，重点建设现代农业示范带，加快发展新能源、特色装备制造、农产品深加工、现代物流产业，建设特色康养旅游度假区，形成产业发展新支撑。实施“多规合一”，逐步构建核心带动、多点支撑、整体推进的城镇化发展新格局。实施乡村振兴战略，发展壮大县域经济，努力实现晋级升位。

3. 坚持绿色发展，在美丽铁岭建设上实现新突破。牢固树立社会主义生态文明观，倡导简约适度、绿色低碳的生产生活方式，推动人与自然和谐发展。实施大气污染防治行动，加强水、土壤、农业面源等污染防治，着力解决环境突出问题。加大生态系统保护力度，创建省级生态县区。继续推进节能减排工作，大力发展循环经济。开展创建节约型机关、绿色家庭、绿色学校、绿色社区、绿色出行等行动，努力建设美丽铁岭。

4. 坚持开放发展，在提升外向型经济发展水平上实现新突破。深入实施开放牵动战略，拓宽开放领域，优化开放环境，打造内陆地区开放新高地。积极融入国家“一带一路”倡议，加强与沈阳经济区、沿海经济带和辽宁自贸区对接，以铁岭保税物流中心为纽带，打造“中国东北陆港”。深入推进与淮安对口合作，加快“铁四通辽”经济协作区建设，推动沈铁工业走廊延伸发展，优化区域开放格局。坚持引进来和走出去并重，持续加大招商引资、招才引智力度，着力引进一批专精特新项目和支撑发展的当家项目。

5. 坚持共享发展，在满足人民美好生活需要上实现新突破。坚持以人民为中心的发展思想，紧紧抓住人民最关心、最直接、最现实的利益问题，努力在幼有所育、学有所教、劳有所得、病有所医、老有所养、住有所居、弱有所扶上取得新进展。坚决打赢防范化解重大风险和精准脱贫攻坚战。全面加强基础设施建设，构建现代综合运输体系。扎实推进平安铁岭建设，加强和创新社会治理，维护社会和谐稳定，确保全市人民安居乐业。

三、2018年工作安排

今年是贯彻党的十九大精神的开局之年，也是加快突破辽西北、决胜全面建成小康社会、推进铁岭振兴发展的关键之年，做好各项工作意义重大。

主要预期目标是：地区生产总值增长7%左右，固定资产投资增长10%以上，一般公共预算收入增长6%，规模以上工业增加值增长7%以上，社会消费品零售总额增长5%，外贸出口总额增长7.8%，城乡常住居民人均可支配收入分别增长7%左右。重点抓好以下几项工作：

（一）深化供给侧结构性改革，推进产业转型升级

继续优化产业结构，促进一二三产业协调发展，大力发展实体经济，支持民营企业加快发展，继续抓好“三去一降一补”，着力在“破、立、降”上下功夫，不断提高经济发展质量和效益。

加快发展现代农业，以现代农业示范带建设为引领，努力打造优质高效的现代农业体系。科学规划建设优质粮食生产功能区、生态规模畜禽养殖区、高效设施园艺展示区、特色农产品优势区和农产品加工集聚区。积极推进国家农业科技示范园区建设。发展特色品牌农业，做强粮食和畜牧两大主导产业，做大榛子、苗木等特色产业规模。抓好润霖、牧原、大北农等规模化养殖项目建设，支持盛德、嬴德、中敖等龙头企业发展，拉长产业链，全面提高畜牧业标准化、规模化、产业化水平。大力发展农业生产性服务业，加大农业基础设施建设力度，提高农业信息化、机械化水平和气象防灾减灾能力。建立和完善支农惠农金融服务体系，强化财政撬动金融作用，引导社会资本服务农业发展。大力培育新型农业经营主体，加快发展多种形式的适度规模经营。

加快构建现代工业发展体系，努力做大工业规模。发展新能源产业，建设调兵山市新能源产业园，抓好风力、光伏、生物质发电项目建设，提高清洁能源比重。发展特色装备制造产业，做大专用车、汽车零部件、通信材料等产业规模，重点发展智能制造、绿色制造和服务型制造，大力推进军民融合产业发展。推进小升规、规转股、大做强，着力培育一批有发展潜力的成长型小微企业，新增产值超亿元企业5户，新增规模以上工业企业20户。发展农产品深加工产业，树立“大食品”理念，加快粮食、肉类、特色产业加工集聚区建设，新增规模以上农业产业化企业8户。嫁接改造停产企业停建项目20个，盘活土地800亩以上。

加快发展现代服务业，提高三产对经济增长的贡献率。发展现代物流产业，着力推进东北城二期、九三恒通物流园二期等项目建设，规划建设凡河新区现代物流基地。建设特色康养旅游度假区，重点推进莲花湿地、蒸汽机车影视小镇等项目开发建设，整合各类旅游资源，加快组建旅游集团，打造精品旅游线路，形成全域旅游网络。推动赢信财富广场、调兵山装饰材料城、红星美凯龙家居广场等大项目建设，提高服务业集聚区发展水平。实施“互联网+流通”行动计划和企业上网工程，推动电子商务进农村、进社区。加快金融广场融资信用平台和网络融资平台建设，完善铁岭银行法人治理结构，完成调兵山市、银州清河农商行组建，积极引进域外金融机构，完善金融服务体系。推进农村普惠金融示范区建设。

（二）深入开展项目年活动，全力以赴抓招商上项目

牢固树立以企业为中心、以项目为支撑的发展理念，把招商引资、招才引智作为“一号工程”，继续开展项目年活动，强化服务保障，以存量稳增长、以增量促发展。

大力引进建设一批项目。着力推动调兵山燃料乙醇二期及5万吨纤维乙醇、昌图古榆树煤层气等一批大项目签约落地，力争富力地产、王千工业园、昌图县30万吨燃料乙醇、益海嘉里扩产、玄武岩纤维及复合材料、中慈产业园、马迭尔冬宫、采煤沉陷区光伏发电等一批新项目开工建设。全年到位内资100亿元以上，实际利用外资2600万美元。

用好用足国家和省扶持政策。包装和储备亿元以上项目100个。争取调兵山一期30万吨燃料乙醇项目投产、铁路物流基地投入运营。加快推进全域化老工业区搬迁改造，尽快启动沈铁城际铁路建设。深入推进与淮安对口合作，抓好“铁四通辽”经济协作区建设。全年向上争取各类政策资金130亿元以上。

深化对外经贸合作。加强对外贸易平台建设，抓好铁岭保税物流中心封关运营。积极拓展对外资源开发和工程承包领域，全力支持铁煤建工越南项目建设。大力扶持外向型企业发展，引进和培育一批出口企业，进一步做大进出口规模。

（三）实施乡村振兴战略，推进城乡协调发展

坚持乡村振兴战略与县域经济发展、新型城镇化建设有机结合、同步推进，加快形成城乡互补、全面融合、共同繁荣的新型城乡发展格局。

大力实施乡村振兴战略。按照产业兴旺、生态宜居、乡风文明、治理有效、生活富裕的总要求，坚持农业、农村、农民并重，生产、生活、生态并进，新体制、新产业、新模式并举，进一步壮大乡村经济实力。积极推进农村集体产权制度和农村承包地“三权分置”改革，基本完成农村承包地确权登记颁证工作，抓好集体林权配套、供销体制和农垦改革，增强农村发展动力，壮大集体经济实力。完善村民自治，推动乡村法治，发展社会主义先进乡村文化，进一步加强基层治理体系和治理能力建设。

加快发展县域经济。推进县域工业园区建设，积极引进新项目，发展新业态，培育新动能。坚持发展理念创新和体制机制创新，按照行政管理轻型化、运行机制企业化、公共服务市场化原则，逐步理顺工业园区管理体制，激发园区发展活力。以资源禀赋为基础，以农产品深加工产业为依托，发展壮大现有企业，着力培育一批铁岭品牌。力争每个县（市）区建设1—2个市级以上特色乡镇或产业特色小镇。加快形成县域“一园一区一镇一品”的发展格局，打造新的经济增长点。

加强基础设施建设。新建和改造国省干线220公里，启动新三线公路三期工程建设，力争辽宁中环铁岭段、沈康高速调兵山连接线工程竣工通车。凡河新区实施人行步道改造6.2万平方米，完成凡河四桥、天水河驳岸等维修改造工程。银州区改造污水泵站4座、管线9条，完成柴河大桥维修改造。开原市重点推进滨水新城7.8平方公里核心区建设。昌图县完善滨湖新城、辽北新城基础和配套设施建设。西丰县推进红旗路贯通和3条过境公路维修改造。调兵山市抓好城市外环公路和供水工程建设。清河区抓好金枫苑、万象新城等项目开发建设。全面实施农网改造，进一步增强供电保障能力。

提升城乡管理水平。推动各项规划有效整合、科学实施。创新城市管理综合执法体制机制，建立城市管理数字化平台，推动智慧铁岭建设。开展城市净化美化行动，对露天市场、占道经营、广告牌匾、机动车乱停乱放等突出问题进行集中治理，中心城区道路清洁率达到100%。抓好生活垃圾分类处理，选择一批公共机构、企业、社区和行政村先行试点。有序推进“厕所革命”，打造省级绿化示范村100个。

（四）扎实推进改革创新，着力增强发展动力和活力

用改革的办法破除体制机制弊端，用创新的举措解决发展中的矛盾和问题，大力推进科技强市、人才兴市工作，激发各类市场主体活力，为经济社会发展增添动力。

全力打造营商环境最优市。继续开展软环境建设年活动。持续深化“放管服”改革，大力实施简政放权，进一步完善投资建设项目审批代办帮办服务，建立预审服务工作制度，推进“互联网+政务服务”建设，用政府权力的减法换取市场活力的加法，用政府服务的高效换取经济发展的高质，用一流的环境推动实现一流的发展。

推进商事制度改革。全面实施“多证合一、一照一码”“先照后证”改革，完善市场准入负面清单和公平竞争审查制度，做好注册登记便利化、全程电子化登记和电子营业执照工作，建立服务企业和群众“最多跑一次”制度。推进政府部门涉企信息归集公示，构建市场信用监管体系。

抓好财税体制改革。积极推进县乡财政体制改革，科学界定事权范围，激发乡镇政府发展经济的内生动力和活力。采取切实可行的激励措施，调动和保护乡镇发展经济、培植财源、组织收入积极性，逐步提升乡镇财政基本保障能力。

强力推进国资国企改革。完善国有资产管理体制，改革国有资本授权经营体制，加快实现国有经济优化布局、结构调整、战略性重组，促进国有资产保值增值，推进国有资本做强做优做大。推进市属经营性国有资产集中统一监管。发展混合所有制经济，加快组建国有企业集团，建立完善法人治理结构。积极推进“三供一业”分离移交，加快推进事业单位改革重组工作步伐，适时启动厂办大集体改革。

全力推进科技创新。围绕产业链部署创新链，围绕创新链安排要素支撑，打通创新成果到产业发展全流程。新发展高新技术企业15户。实施重点产学研合作项目30项。组建省级工程技术中心1个，认定一批市级研发中心和科技型农民合作社。

加强人才队伍建设。实行更加积极、更加开放、更加有效的人才政策，积极培养本土人才，千方百计吸引各级各类人才投入到铁岭振兴发展的事业中。大力弘扬企业家精神、工匠精神和劳模精神，鼓励青年学子回乡创业，让人才的创造活力竞相迸发、聪明才智充分涌流。

（五）始终坚持问题导向，坚决打赢三大攻坚战

瞄准民生、生态建设等关键领域和薄弱环节，精准发力、精准施策，确保取得突破性进展，为决胜全面建成小康社会奠定坚实基础。

打好防范化解重大风险攻坚战。加强政府债务管理，建立债务风险预警机制，加快推进债务置换，加大高风险地区债务化解力度，推进政府融资平台公司市场化转型。防范和化解财政支付风险。千方百计筹措资金，确保养老金按时足额发放。加快建立金融风险防范机制，加强风险评估、监测和应急处理，坚决打击违法违规金融活动，确保地方金融安全。

打好脱贫攻坚战。按照“六个精准”“五个一批”的要求，着力抓好产业扶贫、金融扶贫，落实各项扶贫政策，注重扶贫、扶志和扶智相结合，激发贫困人口内生动力，高质量完成1.7万名建档立卡贫困人口脱贫、65个贫困村销号，西丰贫困县摘帽，确保脱真贫、真脱贫。

打好污染防治攻坚战。认真抓好中央环保督察反馈意见的整改。集中治理燃煤小锅炉、黄标车和老旧车辆，全力推进秸秆综合利用。严格落实河长制，加强水环境综合治理。完成植树造林15.5万亩，辽河干流铁岭段河滨带植被覆盖率、岸线自然保有率均达到100%。落实最严格的耕地保护制度，完成水土流失治理1.1万亩。加强农业面源污染防治，全面推进畜禽养殖废弃物资源化利用，进一步改善人民群众生产生活环境。

（六）加强和创新社会治理，切实保障和改善民生

按照尽力而为、量力而行的原则，努力保障群众基本生活，完善公共服务体系，发展各项社会事业，维护社会和谐稳定。

为全市人民办好15件民生实事。1.实施脱贫攻坚工程。2.扶持创业就业，新增城镇就业1.2万人，确保零就业家庭保持动态为零。3.提高全市城乡最低生活保障标准。4.继续推进新农合异地就医联网即时结报及新农合和大病保险“一站式”报销服务。5.改善40所乡镇中心幼儿园和46所贫困地区义务教育薄弱学校基本办学条件。6.开展送戏下乡、铁岭欢歌、全民读书节等文化惠民活动。7.推进铁岭市综合性老年人养护中心建设。8.建设铁岭市食品安全检验检测中心。9.改造棚户区住房979套。10.维修改造中心城区道路24条。11.维修改造农村道路和一事一议村内道路721公里，努力建设“四好农村路”。12.建设美丽乡村示范村25个。13.综合整治亮子河、马仲河。14.实施贫困残疾人家庭无障碍改造。15.开展困难职工和特殊职业群体帮扶活动。

优先发展教育事业。建设60所连锁幼儿园。开原市和昌图县通过义务教育基本均衡县国家认定，促进县域义务教育城乡一体化优质均衡发展。积极推进普及高中阶段教育，抓好现代职业教育和社会力量办学，加快推进铁岭师专现代服务业专业群项目落实落地。加强师德师风建设，着力解决学生课外负担重、乱补课乱收费和“择校热”“大班额”等突出问题，规范教育教学行为。

加快健康铁岭建设。继续推进城市公立医院改革，加快分级诊疗和医疗联合体建设，鼓励社会资本进入养老、医疗等领域。评选名科名医名护，评定一批优秀乡镇卫生院和社区卫生服务中心。西丰县、清河区启动健康产业和医养结合试点。抓好残疾人精准康复工作。扩大国家级卫生县城创建成果，争创国家级卫生城市和卫生乡镇。大力发展体育事业，广泛开展全民健身活动，增强全民健康素质。

推动文化事业和产业发展。大力弘扬社会主义先进文化，组织好纪念改革开放40周年文艺活动。推进东北城文化产业园等文化产业示范基地建设，各县（市）区分别培育一个文化产业品牌。进一步繁荣文艺创作，推出一批文艺精品。加大少数民族非物质文化遗产保护力度，打造一批民族特色村镇，争创国家和省级少数民族特色村寨。培育和践行社会主义核心价值观，广泛开展群众性精神文明创建活动，加快基层综合文化服务中心和文化广场建设，开展道德模范、身边好人等评选活动，讲述铁岭好故事，传播文明正能量。

加强和创新社会治理。开展“七五”普法，全面落实“谁执法谁普法”责任制。推行社区治理体系和“一村（社区）一法律顾问”制度，实现政府治理、社会调节和居民自治的良性互动。加强信访、市长服务热线和民心网诉求办理工作。强化基层应急预案编制和应急演练，增强国防动员系统的组织动员力、快速反应力和支援保障力。加强全民国防教育，进一步做好拥军优属工作。落实安全生产责任制，推进风险防控与隐患排查治理双重预防机制建设，坚决防范重特大安全生产事故。开展食品药品专项整治，争创食品安全城市。积极推进平安铁岭建设，建立健全立体化社会治安防控体系，稳步提升群众安全感和满意度。

四、努力建设人民满意的服务型政府

使命引领担当，使命引领未来。本届政府肩负着决胜全面建成小康社会、推进铁岭振兴发展的历史重任。我们一定要坚守造福人民的宗旨，强化奋发有为的使命担当，进一步提振干事创业的士气，鼓足敢于担当的勇气，强化不甘落后的志气，培育清正廉明的正气，凝聚上下同欲的人气，努力开创政府工作新局面。

始终强化政治建设。深入学习贯彻党的十九大精神，坚持用习近平新时代中国特色社会主义思想武装头脑、指导实践、推动工作。牢固树立“四个意识”，旗帜鲜明讲政治，坚决维护以习近平同志为核心的党中央权威和集中统一领导，始终在思想上政治上行动上同党中央保持高度一致。确保党中央、国务院和省委、省政府的各项决策部署，在铁岭大地落地生根、开花结果。

全面推进依法行政。牢固树立法治理念，依法履行政府职责。坚决维护市委领导，始终与市委同心、同向、同力。自觉接受市人大及其常委会的法律监督和工作监督，认真落实市人大及其常委会的各项决议决定。加强同人民政协的联系，自觉接受市政协的民主监督。积极推行政府法律顾问制度，深化监察体制改革，加强审计和统计工作，完善决策程序，不断提高政府工作法治化、科学化、规范化水平。

坚定不移转变作风。大力弘扬“干”字当头、“实”字为先、“成”字为要的精神，哈下腰来、扑下身子，倾注真情用心干、撸起袖子加油干、甩开膀子奋力干，以功成不必在我、成功必定有我的胸怀，不做表面文章，不搞短期行为，既要打好攻坚战，又要打好持久战，用钉钉子的精神做实做细做好各项工作，以实实在在的工作成效和发展业绩取信于民。

驰而不息正风肃纪。打铁必须自身硬。严格落实全面从严治党主体责任，切实履行“一岗双责”。深入落实中央八项规定精神和省委实施意见，持续整治“四风”问题，扎实做好巡视反馈意见的整改和落实。严肃党内政治生活，认真开展“不忘初心、牢记使命”主题教育。切实加强政府自身建设，不断增强政府的执行力和公信力。进一步建立健全容错纠错机制，为干事者担当，为尽责者负责，充分调动全市广大干部群众的积极性、主动性和创造性，形成推动铁岭振兴发展的磅礴力量。

各位代表，新时代赋予新使命，新征程要有新作为。让我们乘着党的十九大东风，更加紧密地团结在以习近平同志为核心的党中央周围，高举习近平新时代中国特色社会主义思想伟大旗帜，在中共铁岭市委的坚强领导下，进一步解放思想，开拓创新，埋头苦干，为决胜全面建成小康社会、实现铁岭振兴发展而努力奋斗！
